# Rabbit xenogeneic transplantation model for evaluating human chondrocyte sheets used in articular cartilage repair

**DOI:** 10.1002/term.2741

**Published:** 2018-08-14

**Authors:** Takumi Takahashi, Masato Sato, Eriko Toyoda, Miki Maehara, Daichi Takizawa, Hideyuki Maruki, Ayako Tominaga, Eri Okada, Ken Okazaki, Masahiko Watanabe

**Affiliations:** ^1^ Department of Orthopaedic Surgery, Surgical Science Tokai University School of Medicine Isehara Japan; ^2^ Department of Orthopaedic Surgery Tokyo Women's University Tokyo Japan

**Keywords:** allogeneic transplantation, articular, cartilage, chondrocytes, preclinical, transplantation, xenogeneic transplantation

## Abstract

Research on cartilage regeneration has developed novel sources for human chondrocytes and new regenerative therapies, but appropriate animal models for translational research are needed. Although rabbit models are frequently used in such studies, the availability of immunocompromised rabbits is limited. Here, we investigated the usefulness of an immunosuppressed rabbit model to evaluate directly the efficacy of human chondrocyte sheets through xenogeneic transplantation. Human chondrocyte sheets were transplanted into knee osteochondral defects in Japanese white rabbits administered with immunosuppressant tacrolimus at a dosage of 0.8 or 1.6 mg/kg/day for 4 weeks. Histological evaluation at 4 weeks after transplantation in rabbits administered 1.6 mg/kg/day showed successful engraftment of human chondrocytes and cartilage regeneration involving a mixture of hyaline cartilage and fibrocartilage. No human chondrocytes were detected in rabbits administered 0.8 mg/kg/day, although regeneration of hyaline cartilage was confirmed. Histological evaluation at 12 weeks after transplantation (i.e., 8 weeks after termination of immunosuppression) showed strong immune rejection of human chondrocytes, which indicated that, even after engraftment, articular cartilage is not particularly immune privileged in xenogeneic transplantation. Our results suggest that Japanese white rabbits administered tacrolimus at 1.6 mg/kg/day and evaluated at 4 weeks may be useful as a preclinical model for the direct evaluation of human cell‐based therapies.

## 1 INTRODUCTION

Articular cartilage is a complex tissue capable of withstanding repetitive forces experienced by the knee joint through daily movements such as walking and stair climbing. Because of its complexity and avascular nature, articular cartilage is prone to irreversible damage from trauma or aging that often progresses to degenerative conditions such as osteoarthritis (OA) (Brittberg et al., 2016). OA is a debilitating disease that limits mobility and imposes a major social and healthcare burden, especially in ageing populations. The increases in longevity and size of the elderly population mean that more people are living longer with disability and a reduced quality of life (Ondrésik et al., 2017).

Therapies for restoring normal knee function through the regeneration of articular cartilage have been investigated (Mardones, Jofré, & Minguell, 2015), but the development of an effective treatment for OA is still a work in progress. To date, bone marrow‐stimulation techniques (Erggelet & Vavken, 2016); autologous and allogeneic osteochondral grafts (Bugbee, Pallante‐Kichura, Görtz, Amiel, & Sah, 2016; Hangody & Füles, 2003); and autologous chondrocyte implantation (Brittberg et al., 1994; Clavé et al., 2016; Tohyama et al., 2009) have been used for the treatment of relatively small cartilage defects. The clinical outcomes so far suggest some success, but evidence is inconclusive, and there are concerns about repair with fibrocartilage and donor site morbidity (Matricali, Dereymaeker, & Luyten, 2010).

Novel therapies being developed for articular cartilage repair can be categorized as scaffold only, cells with a scaffold, or cells without a scaffold (scaffoldless; Kon, Roffi, Filardo, Tesei, & Marcacci, 2015). Our approach has been to use cell sheet technology developed in the 1990s (Kikuchi, Okuhara, Karikusa, Sakurai, & Okano, 1998; Okano, Yamada, Okuhara, Sakai, & Sakurai, 1995; Yamada et al., 1990) to culture chondrocytes on temperature‐responsive culture inserts coated with poly(N‐isopropylacrylamide), which allows the collection of chondrocytes as cell sheets without disrupting the extracellular matrix. Using this scaffoldless technology, we have reported the usefulness of layered chondrocyte sheets (Kokubo et al., 2016) in the treatment of partial‐thickness defects in rabbits (Kaneshiro et al., 2006) and full‐thickness defects in rats (Takaku et al., 2014); rabbits (Ito et al., 2012); and minipigs (Ebihara et al., 2012). In a clinical study from 2011 to 2014, we treated eight patients with autologous chondrocyte sheets and confirmed the safety and effectiveness of this method in treating patients with OA (Sato, Yamato, Hamahashi, Okano, & Mochida, 2014).

However, autologous transplantation requires two surgeries, and the cell source is often limited. Thus, we are currently investigating the possibility of creating allogeneic chondrocyte sheets. For cartilage regeneration, both differentiated (Ham et al., 2015) and undifferentiated mesenchymal stem cells (Richardson et al., 2016) from various sources such as bone marrow, adipose tissue, and synovial tissue have been investigated as potential sources. Hyaline cartilaginous tissue generated from embryonic stem cells (Oldershaw et al., 2010) and, more recently, from induced pluripotent stem cells (Yamashita et al., 2015) shows great potential. To address the feasibility and traceability issues, we have been investigating the use of chondrocytes obtained from young polydactyly patients (Nasu, Takayama, & Umezawa, 2016) in preparation for a first‐in‐human clinical study for allogeneic chondrocyte sheets that has been approved by the Ministry of Health, Labour and Welfare of Japan.

The main objective of this study was to establish a preclinical animal model that would allow the direct evaluation of human chondrocyte sheets for translational research purposes. Rabbits are phylogenetically and anatomically more closely related to humans than rodents (Schnupf & Sansonetti, 2012), and the size of defects that can be created allows for the transplantation of a single chondrocyte sheet without overpopulating the defect. Human adult chondrocyte sheets were fabricated from surgical remains of total knee arthroplasty (TKA) and transplanted into osteochondral defects in Japanese white (JW) rabbits immunosuppressed by tacrolimus, a potent immunosuppressant used in organ transplantation (Ikebe et al., 1996; Kino et al., 1987). We hypothesized that a xenogeneic transplantation rabbit model would be a cost‐effective method for the direct evaluation of the in vivo efficacy of human chondrocyte sheets.

## 2 MATERIALS AND METHODS

The animal experiments were approved by the Institutional Animal Experiment Committee at Tokai University and were performed in accordance with the guidelines of the Institutional Regulation for Animal Experiments and the Fundamental Guideline for Proper Conduct of Animal Experiment and Related Activities in Academic Research Institutions under the jurisdiction of the Ministry of Education, Culture, Sports, Science, and Technology for animal handling and care.

### 2.1 Fabrication and analysis of TKA sheets

Under the approval and guidance of the Tokai University Ethics Committee and with patients' informed consent, cartilage and synovial tissues were obtained from five patients (age 61–80 years; average age 71.4 years) who underwent TKA at Tokai University Hospital. Chondrocytes and synovial cells were enzymatically isolated, and chondrocyte sheets made from cells obtained from TKA (TKA sheets) were prepared separately from each donor using the coculture method as previously described (Kokubo et al., 2016). Briefly, cartilage tissue and synovial tissue were separately minced and digested with 5 mg/ml collagenase Type I (Worthington Biochemical Corp., Lakewood, NJ, USA) in Dulbecco's modified Eagle's medium–Nutrient Mixture F‐12 (DMEM/F12; Gibco, Waltham, MA, USA) supplemented with 20% fetal bovine serum (FBS; Ausgenex, Molendinar, Australia) and 1% antibiotic–antimycotic solution (AA; Gibco). The cells were filtered through a 100‐μm cell strainer (Becton, Dickinson, and Company [BD], Franklin Lakes, NJ, USA) and washed in phosphate‐buffered saline (PBS; Gibco). Primary chondrocytes were stored at −80°C in Cellbanker 1 cryopreservation medium (Zenoaq, Fukushima, Japan), and synovial cells were cultured to Passage 1 and then stored similarly.

To fabricate TKA sheets, synovial cells at Passage 1 were first plated as feeder cells in six‐well dishes (BD) at a density of 1 × 10^4^ cells per cm^2^ with 3 ml of medium. After 1 hr, temperature‐responsive culture inserts (Cellseed, Tokyo, Japan) were placed in the wells, and primary chondrocytes were seeded without direct contact to synovial cells at a density of 5 × 10^4^ cells per cm^2^ with 2 ml of medium. The medium was changed every 3 or 4 days thereafter with the addition of 100 μg/ml ascorbic acid (Wako Pure Chemical Industries, Osaka, Japan). At 2 weeks, three sheets of only chondrocytes were layered using a support membrane of polyvinylidene difluoride, and the layered sheets were cultured for another 7 days.

To count the cells, fabricated TKA sheets were digested enzymatically and stained with trypan blue. For histological analysis, TKA sheets were fixed in 4% paraformaldehyde in phosphate buffer and embedded in optimal cutting temperature compound (Sakura Finetek Japan, Tokyo, Japan). Twenty‐micrometre‐thick sections were stained with haematoxylin and eosin (HE) or with Safranin O, Fast Green, and HE. For immunohistochemical analysis, 10‐μm sections were blocked with 5% normal goat serum (NGS; Rockland Immunochemicals, Limerick, PA, USA) and 0.3% Triton X‐100 (Sigma‐Aldrich, St. Louis, MO, USA) in phosphate buffer for 30 min. The sections were then incubated with primary antibodies to human Type I collagen (COL1; SouthernBiotech, Birmingham, AL, USA; dilution 1:200); Type II collagen (COL2; Kyowa Pharma Chemical, Toyama, Japan; dilution 1:200); aggrecan (R&D Systems, Minneapolis, MN, USA; dilution 1:10); or fibronectin (Merck Millipore, Darmstadt, Germany; dilution 1:500) at 4°C overnight. The sections were washed and incubated at room temperature (RT) for 1 hr with the secondary antibody Alexa Fluor 488‐conjugated goat anti‐mouse immunoglobulin (Ig; Thermo Fisher Scientific, Waltham, MA, USA) for COL2 and fibronectin, and Alexa Fluor 488‐conjugated anti‐goat Ig (Thermo Fisher Scientific) for COL1 and aggrecan. The slides were then stained with 4′,6‐diamidino‐2‐phenylindole (Vector Laboratories, Burlingame, CA, USA) and observed under a BZ‐9000 Generation II fluorescence microscope (Keyence Corp., Osaka, Japan).

To measure the concentrations of humoral factors produced by TKA sheets, fabricated sheets were placed in DMEM/F12 supplemented with 1% FBS and 1% AA, and the culture supernatants were collected after 72 hr, centrifuged at 15,885 *g* for 5 min to remove debris, and stored at −80°C. Commercial enzyme‐linked immunosorbent assays were used to quantify the concentrations of transforming growth factor‐β1 (R&D Systems) and melanoma‐inhibitory activity (MIA; Roche, Basel, Switzerland).

### 2.2 Measurement of blood tacrolimus concentration in JW rabbits

All JW rabbits were purchased from Tokyo Laboratory Animals Science Co. (Tokyo, Japan). The blood concentration of tacrolimus was monitored in three female JW rabbits (average weight = 3.0 kg) independent from the transplantation experiment. Tacrolimus (Astellas Pharma, Tokyo, Japan) was administered daily for 14 days intramuscularly at a dosage of 1.6 mg/kg/day. Blood samples from the ear were collected into EDTA 2 K tubes (Tokuyama Sekisui Co., Yamaguchi, Japan); frozen at −30°C; and sent to SRL Inc. (Tokyo, Japan) for analysis. Tacrolimus concentration in blood was measured using an electrochemiluminescence immunoassay on a Cobas e 411 immunoassay analyser (Hitachi High Technologies Co., Tokyo, Japan) with a minimum detection level of 0.5 ng/ml.

### 2.3 Transplantation of TKA sheets

Thirty female JW rabbits (average weight = 3.0 kg) were used in the transplantation experiment. The animals were housed one animal per cage and were given daily standard chow and access to water ad libitum. Before surgery, rabbits were randomly assigned by weight to one of five groups: A (defect only, 4 weeks, 1.6 mg/kg/day of tacrolimus); B (TKA sheet, 4 weeks, 0.8 mg/kg/day); C (TKA sheet, 4 weeks, 1.6 mg/kg/day); D (defect only, 12 weeks, 1.6 mg/kg/day); and E (TKA sheet, 12 weeks, 1.6 mg/kg/day). TKA sheets fabricated from each of the donors were equally allocated to each transplantation group. Tacrolimus was administered intramuscularly daily for 10 days starting 2 days before transplantation and then every other day until 4 weeks after surgery. The intramuscular injections alternated between the right and left hind legs.

For surgery and transplantation, the rabbits were anaesthetized with 2 L/min nitrous oxide, 1 L/min oxygen, and 2.5–3.0% isoflurane (Pfizer, New York City, NY, USA). A medial parapatellar incision was made to the right knee, and the patella was dislocated to access the patellar groove of the femur. A 5‐mm biopsy punch (Kai Industries, Gifu, Japan) was used as a marking guide, and a 5‐mm drill was used to create an osteochondral defect (diameter = 5 mm; depth = 3 mm). Slight bleeding from the subchondral bone was confirmed, and physiological saline (Nipro, Osaka, Japan) was used to clean the defect and prevent thermal damage. For transplantation groups B, C, and E, one TKA sheet was transplanted into each defect without suturing. After restoration of the patella, the quadriceps femoris muscle and tendon were sutured to prevent dislocation.

### 2.4 Monitoring of biochemical markers in blood

Blood monitoring was performed weekly for selected rabbits (*n* = 3 for each group) in defect group A and transplantation group C from Day 0 (before surgery) to Day 28 (before euthanasia). Blood samples were collected from the ear, placed in EDTA 2 K tubes and BD Vacutainer SST II Advance tubes, and frozen at −30°C. Samples were sent to Fujifilm Monolith Co. (Tokyo, Japan) for analysis. Abnormalities in blood chemistry were monitored, especially to detect changes in kidney and liver function.

### 2.5 Pain evaluation

The Linton Incapacitance Tester (Linton Instrumentation, Diss, Norfolk, England) was used to evaluate the degree of pain, inflammation, or discomfort, as previously reported (Ito et al., 2012). Measurements were made before surgery, on Days 1, 4, 7, 10, 14, 17, 21, 24, and 28 for the first 4 weeks, and on Days 35, 42, 49, 56, 70, and 84 for the following 8 weeks. The average damaged limb weight distribution ratio (%) of the hind limbs was calculated from 10 repeated measurements for each animal and averaged for all groups as follows.

$$\text{Damaged limb weight distribution ratio}\;\left(\%\right)=\frac{\text{Damaged limb load}\;\left(g\right)}{\text{Total limb load}\;\left(g\right)}\times 100$$

### 2.6 Histological evaluation of regenerated cartilage

Rabbits were euthanized by an intravenous administration of 50 mg/ml pentobarbital (Tokyo Chemical Industry, Tokyo, Japan) at 4 weeks or 12 weeks. The operated knee was opened, and the distal portion of the femur was excised and fixed in 20% formalin (Wako Pure Chemical Industries) for 3–5 days. The sample was decalcified in 10% EDTA (Wako Pure Chemical Industries) for 3–4 weeks and embedded in paraffin wax, and 3‐μm sections were cut near the centre of the defect area, parallel to the long axis of the femur.

Standard protocols were used for histological staining. Deparaffinized sections were stained with HE only or with Safranin O, Fast Green, and HE. Safranin O‐stained sections were randomized and scored separately by two trained orthopaedic surgeons (H. M. and D. T.), who were blinded to their identity, using a modified version of the O'Driscoll score and International Cartilage Repair Society (ICRS) score (Mainil‐Varlet et al., 2003; O'Driscoll, Keeley, & Salter, 1986).

To immunostain for COL1 and COL2, deparaffinized sections were treated with 0.4% pepsin (Agilent, Santa Clara, CA, USA) for 30 min at 37°C. The sections were washed in distilled water, treated with 0.3% hydrogen peroxide–methanol solution at RT for 15 min, washed in PBS, blocked with 2.5% NGS for 10 min at RT, and then treated for 3 hr at RT with mouse monoclonal antibody to either human COL1 or human COL2 (Kyowa Pharma Chemical Co., Toyama, Japan) diluted at 1:100 with 1% bovine serum albumin (Sigma‐Aldrich) in PBS. The stained sections were washed in PBS, treated for 1 hr at RT with ImmPRESS polymer anti‐mouse IgG reagent (Vector Laboratories), immersed for 2–8 min in Tris–HCl buffer (pH 7.6) containing 0.02% diaminobenzidine and 0.005% hydrogen peroxide, and then counterstained with HE.

To immunostain for human vimentin, deparaffinized sections were treated with 10‐mM sodium citrate buffer (pH 6.0) for 10 min at 98°C in a microwave. The sections were cooled for 30 min, washed in PBS, and then treated with 5% NGS, followed by Alexa Fluor 647‐conjugated rabbit monoclonal antibody to human vimentin (Cell Signaling Technology, Danvers, MA, USA) diluted at 1:100 with 1% bovine serum albumin in PBS overnight at 4°C. Sections were washed in distilled water and then mounted and cured with 4′,6‐diamidino‐2‐phenylindole (Vector Laboratories) according to the manufacturer's instructions.

All microscopic images were obtained using a BZ‐9000 Generation II fluorescence microscope (Keyence Corp.).

### 2.7 Statistical analysis

Numerical results are expressed as mean and standard deviation unless otherwise noted. ICRS scores are expressed as mean and standard error of the mean. Repeated measures analysis of variance was used to analyse measurements from the monitoring of biochemical makers in blood. Analysis of variance was used to analyse ICRS scores, and Tukey's honest significance test was used for post hoc analysis. The weight distribution ratios were compared with values before surgery using the paired *t* test.

## 3 RESULTS

### 3.1 Properties of TKA sheets

**Figure 1:**
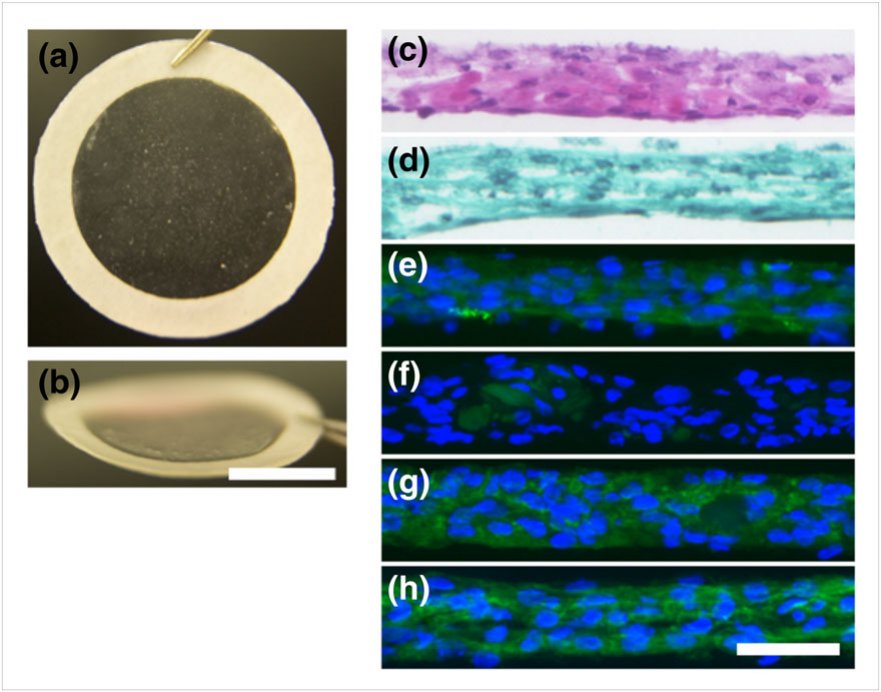
Representative macrographs and micrographs of TKA sheets. (a) Macrograph of a TKA sheet attached to a PVDF support membrane and (b) the same thin sheet seen from an angle. Scale bar = 1 cm. Histological analysis of sections of layered chondrocyte sheets stained with (c) HE and (d) Safranin O. Immunohistochemical analysis revealed (e) positive staining for COL1, (f) slight staining for COL2, (g) positive staining for ACAN, and (h) positive staining for FN. Scale bar = 50 μm. ACAN: aggrecan; COL1: Type I collagen; COL2: Type II collagen; FN: fibronectin; HE: haematoxylin and eosin; TKA: total knee arthroplasty; PVDF: polyvinylidene difluoride

An average TKA sheet contained 1.6 ± 0.2 × 10^6^ cells and had a thickness of 50.0 ± 6.5 μm. The sheets were layered and manipulated using a polyvinylidene difluoride support membrane, which was removed upon transplantation (Figure 1a,b). HE staining of TKA sheets showed the integration of the three chondrocyte sheet layers and the multilayer of chondrocytes 1 week after layering (Figure 1c). TKA sheets stained negative for Safranin O (Figure 1d), positive for COL1 (Figure 1e), slightly positive for COL2 (Figure 1f), positive for aggrecan (Figure 1g), and positive for fibronectin (Figure 1h). Enzyme‐linked immunoassays showed that an average TKA sheet produced 1.8 ± 0.2 ng/ml of transforming growth factor‐β1 and 14.3 ± 2.1 ng/ml of MIA in 3 ml of culture media in 72 hr.

### 3.2 Blood tacrolimus concentration in JW rabbits

**Figure 2:**
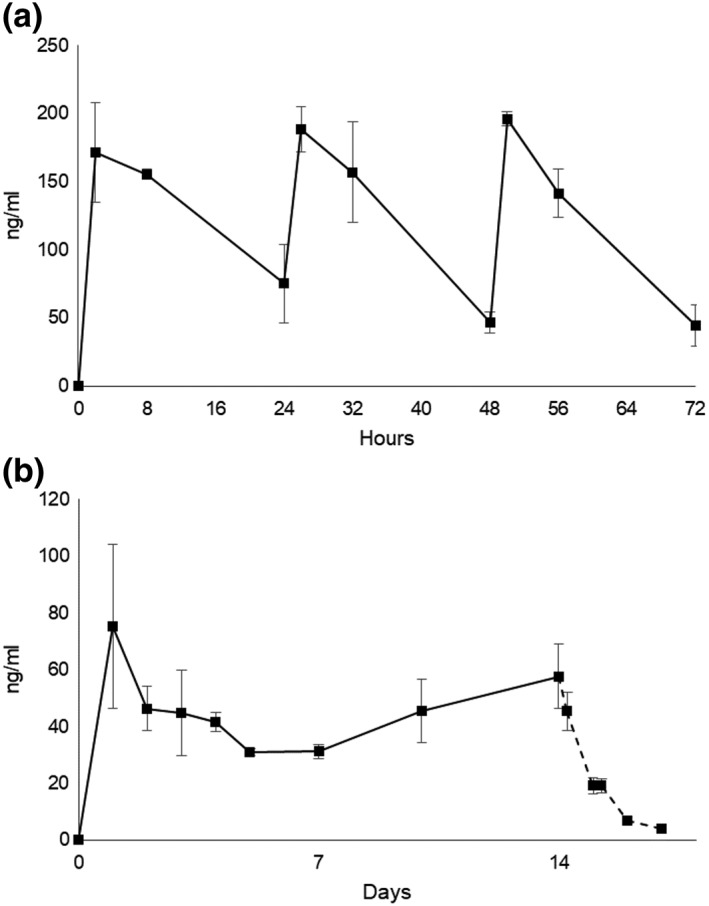
Blood tacrolimus concentration (ng/ml) in Japanese white rabbits (*n* = 3) administered 1.6 mg/kg/day intramuscularly from Days 0 to 13. (a) Measurements were made at 2, 8, and 24 hr after injection for the first 3 days. (b) Trough levels were measured at 24 hr after injection just before the next dose for 14 days. Additional measurements on Days 15, 16, and 17 indicated the metabolism of tacrolimus after the end of the injections

The blood tacrolimus concentration (ng/ml) in three JW rabbits administered 1.6 mg/kg/day for 14 days was monitored for 17 days. Peak concentration measured 2 hr after administration on Days 1, 2, and 3 was 171.5 ± 36.5, 188.5 ± 16.5, and 196.0 ± 5.0, respectively (Figure 2a). The trough concentration was measured at 24 hr after injection and was highest on Day 1 (75.3 ± 28.8) and lowest on Days 5 (31.0 ± 1.4) and 7 (31.2 ± 2.4). The blood tacrolimus concentration increased on Days 10 (45.5 ± 11.3) and 14 (57.6 ± 11.3). After the final injection on Day 14, the concentration continued to decrease to Day 17 (4.0 ± 0.9; Figure 2b). After the immunosuppression was terminated, the animals' appetite returned to normal, and body weight increased gradually.

### 3.3 Xenogeneic transplantation of TKA sheets in immunosuppressed JW rabbits

The surgeries were uneventful, and the TKA sheets fully covered the defect areas. Loss of appetite and diarrhoea were observed after surgery, and a subsequent decrease in body weight was observed; largest decrease in average weight for each group was as follows: 0.16 kg at Day 14 in Group A; 0.32 kg in Group B at Day 21; 0.09 kg in Group C at Day 21; 0.24 kg in Group D at Day 10; and 0.13 kg in Group E at Day 4. Adverse events were detected near the end of the 4 weeks in two rabbits from Group C and in four rabbits from Group B. Self‐inflicted wounds to the end of the hind limbs were observed, but no abnormalities to the surgical areas were detected. Adverse events were detected in two rabbits from Group E. Self‐inflicted wounds to the end of the hind limbs were observed in one rabbit, and swelling in the surgical knee joint was detected in the other. In all rabbits, muscle stiffness and muscle loss were observed in areas where tacrolimus had been administered.

### 3.4 Monitoring of biochemical markers in blood

Blood monitoring was performed weekly for selected rabbits (*n* = 3) in defect group A and transplantation group C (Table 1) starting from Day 0 before transplantation. Creatinine concentration remained within the standard physiological level in the defect group (0.7–1.0 mg/dl) and transplantation group (0.7–0.9 mg/dl). Blood urea nitrogen level also remained within the standard physiological level in the defect group (11.7–18.0 mg/dl) and transplantation group (12.5–19.0 mg/dl). No indications of liver or kidney failure were observed. Creatine phosphokinase level increased on Day 7 in the transplantation group, and C‐reactive protein level increased on Days 7 and 14 in both groups, but these decreases may have reflected the muscle damage caused by the surgery and intramuscular administration. No significant differences were detected between the two groups for the measured biochemical markers.

**Table 1:** Monitoring of biochemical markers in blood

	*p* value	Defect group A					Transplantation group C				
Day		0	7	14	21	28	0	7	14	21	28
Albumin (g/dl)	0.141	4.5	4.0	4.3	4.5	4.4	4.6	4.3	4.4	4.7	4.4
Total bilirubin (mg/dl)	1.000	0.1	0.1	0.1	0.1	0.1	0.1	0.1	0.1	0.1	0.1
AST (IU/l)	0.836	23.7	21.7	19.7	17.7	17.7	22.0	23.0	18.0	13.5	20.5
ALT (IU/l)	0.701	50.0	59.0	52.3	38.3	36.3	63.0	53.5	55.5	40.5	39.0
CPK (IU/l)	0.117	1470	1740	1294	842	893	708	4159	1836	895	1688
BUN (mg/dl)	0.238	14.3	18.7	11.7	13.7	18.0	17.0	19.0	12.5	15.5	16.5
Creatinine (mg/dl)	0.687	1.0	0.8	0.7	0.8	0.8	0.9	0.8	0.7	0.8	0.8
Uric acid (mg/dl)	0.163	0.1	0.1	0.1	0.1	0.1	0.3	0.2	0.1	0.2	0.2
Sodium (mEq/l)	0.504	143	141	142	143	144	139	142	141	141	142
Chloride (mEq/l)	0.354	103	104	102	104	107	105	102	104	105	106
Potassium (mEq/l)	0.508	3.4	4.3	4.2	4.9	5.1	3.9	4.4	4.2	4.4	4.8
Blood glucose (mg/dl)	0.760	111	151	123	125	113	112	147	127	128	122
CRP (mg/dl)	0.923	0.4	10.5	5.3	1.3	0.4	0.4	6.5	6.3	0.7	0.6

*Note*. ALT: alanine transaminase; AST: aspartate aminotransferase; BUN: blood urea nitrogen; CPK: creatine phosphokinase; CRP: C‐reactive protein.

### 3.5 Pain evaluation

**Figure 3:**
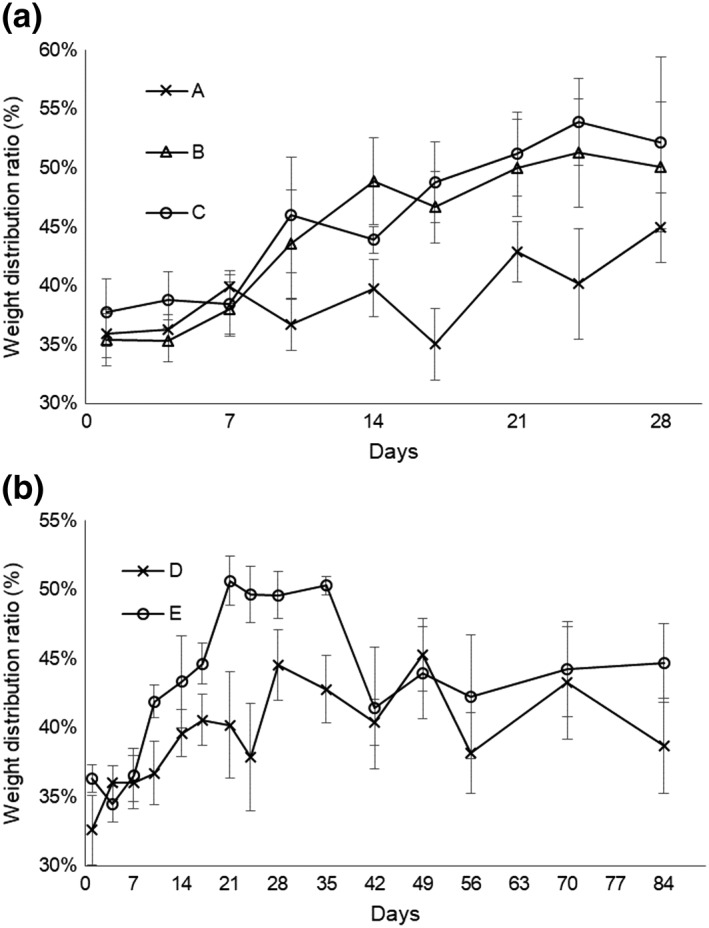
Change in weight distribution ratio determined as the ratio between the operated hind limb load and the total hind limb load. (a) Group A: defect only, 4 weeks, 1.6 mg/kg/day of tacrolimus; (b) Group B: total knee arthroplasty (TKA) sheet, 4 weeks, 0.8 mg/kg/day; (c) Group C: TKA sheet, 4 weeks, 1.6 mg/kg/day; (d) Group D: defect only, 12 weeks, 1.6 mg/kg/day; and (e) Group E: TKA sheet, 12 weeks, 1.6 mg/kg/day. (a) The ratio recovered to the value before surgery in transplantation groups B (*p* = 0.972) and C (*p* = 0.214) by Day 21 but never fully recovered by Day 28 in defect group A (*p* = 0.008). (b) The ratio recovered to the value before surgery by Day 21 in transplantation group E (*p* = 0.593) but worsened at Day 42 (*p* = 0.010) and never recovered fully by Day 84 in Group D (*p* = 0.015)

The weight distribution ratio was used as a measure of pain and was followed for 4 weeks (Figure 3a) and 12 weeks (Figure 3b). This ratio recovered to the value before surgery by Day 21 in transplantation groups B (*p* = 0.972) and C (*p* = 0.214) but did not return fully to the value before surgery by Day 28 in defect group A (*p* = 0.008; Figure 3a). The results of the 12‐week evaluation are shown in Figure 3b. The weight distribution ratio recovered to the value before surgery by Day 21 in transplantation group E (*p* = 0.593) but worsened at Day 42 (*p* = 0.010) and did not recover thereafter. This ratio did not recover fully by Day 84 in defect group D (*p* = 0.015).

### 3.6 Macroscopic and microscopic analysis of regenerated cartilage

**Figure 4:**
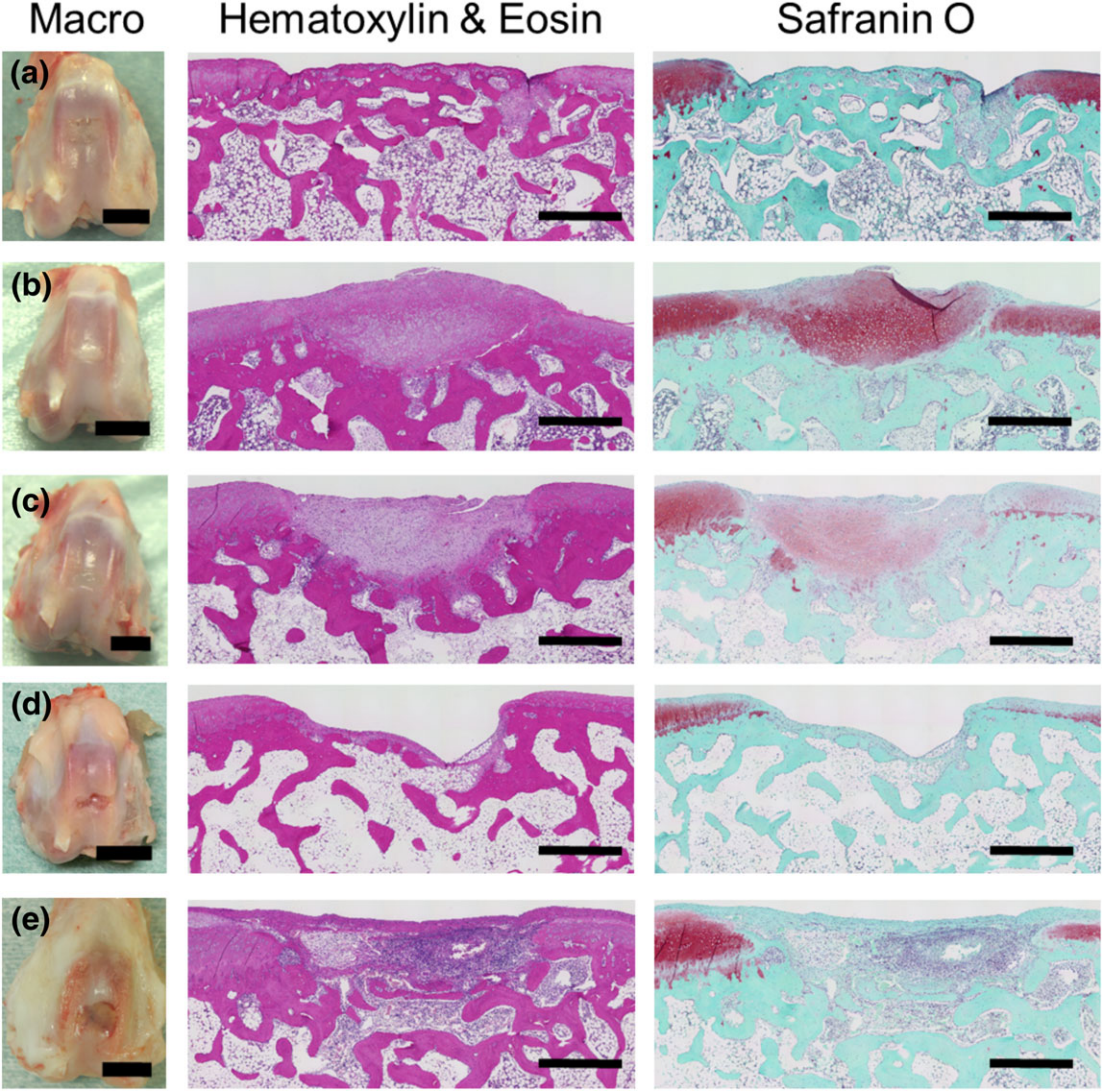
Representative macroscopic images of the defect areas in the patellar groove of the femur and representative microscopic images from the histological analysis of paraffin sections of the defect area. (a) Group A: defect only, 4 weeks, 1.6 mg/kg/day of tacrolimus; (b) Group B: total knee arthroplasty (TKA) sheet, 4 weeks, 0.8 mg/kg/day; (c) Group C: TKA sheet, 4 weeks, 1.6 mg/kg/day; (d) Group D: defect only, 12 weeks, 1.6 mg/kg/day; and (e) Group E: TKA sheet, 12 weeks, 1.6 mg/kg/day. Macroscopically, at 4 weeks, the defect was (a) filled with irregular tissue and (b, c) filled with white smooth material. At 12 weeks, the defect was (d) unfilled and (e) unfilled and showing signs of severe synovial fluid accumulation and inflammation. Left scale bars = 1 cm. Histological analysis revealed (a) subchondral bone filling in part of the defect area. (b) Strong staining for Safranin O was observed. (c) Slight staining for Safranin O was observed. (d) The defect area was unfilled, and Safranin O staining was weak for surface areas surrounding the defect area. (e) Inflammatory cells filled the defect area including parts of the subchondral bone, and no staining for Safranin O was observed in the defect area. Middle and right scale bars = 1 mm

Representative macroscopic images of the defect area for each group are shown in Figure 4. The images show filling of the defect by smooth white tissue in transplantation groups B (Figure 4b) and C (Figure 4c). By contrast, the defect areas were either unfilled or partially filled with irregular tissue in defect groups A (Figure 4a) and D (Figure 4d). In transplantation group E, the defect areas were filled with synovial fluid and showed attachment of surrounding tissue to the synovium and indications of inflammation (Figure 4e).

HE and Safranin O staining was performed to evaluate the regenerated cartilage, as shown in Figure 4. Group A showed little Safranin O staining or an increase in subchondral bone filling in the defect area (Figure 4a). Group B showed strong Safranin O staining (Figure 4b), whereas Group C showed weak Safranin O staining (Figure 4c). Group D showed weak Safranin O staining for surface areas surrounding the defect area. Group E showed signs of inflammatory cells within the defect area and subchondral bone, and no regeneration of articular cartilage (Figure 4e).

**Figure 5:**
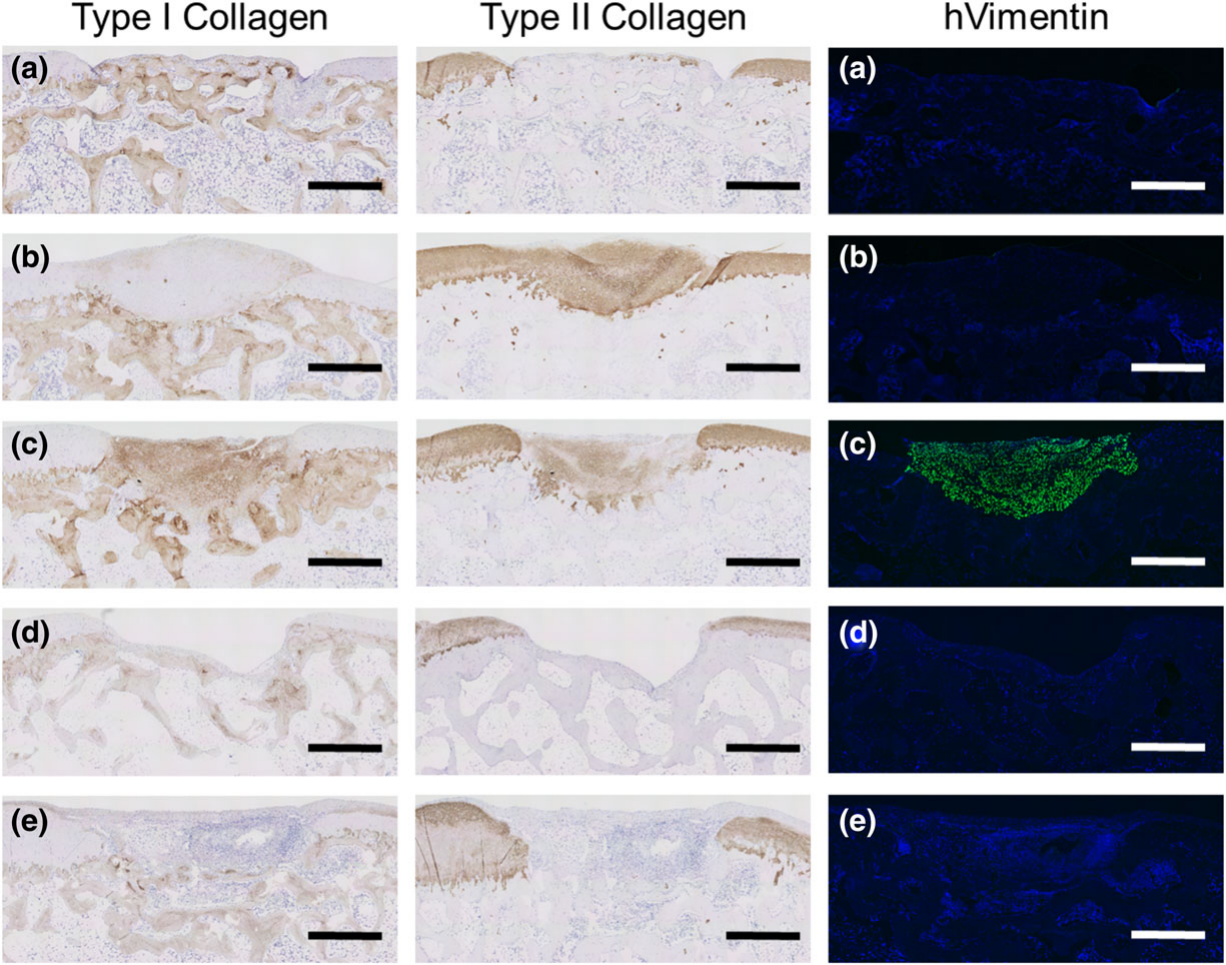
Representative microscopic images from the immunohistochemical analysis of paraffin sections of the defect area. (a) Group A: defect only, 4 weeks, 1.6 mg/kg/day of tacrolimus; (b) Group B: total knee arthroplasty (TKA) sheet, 4 weeks, 0.8 mg/kg/day; (c) Group C: TKA sheet, 4 weeks, 1.6 mg/kg/day; (d) Group D: defect only, 12 weeks, 1.6 mg/kg/day; and (e) Group E: TKA sheet, 12 weeks, 1.6 mg/kg/day. Sections were stained for Types I and II collagens and for human‐specific vimentin (hVimentin). (a) Positive for Type I collagen but negative for Type II collagen. (b) Strong staining for Type II collagen but negative for Type I collagen and hVimentin. (c) Staining for both Types I and II collagens and for hVimentin in the entire defect area. (d) The defect area was unfilled. (e) Inflammatory cells filled the defect area, which was negative for Types I and II collagens and hVimentin. Scale bars = 1 mm

Immunohistochemical analysis was used to evaluate the regenerated cartilage (Figure 5). Samples from transplantation group B stained strongly for COL2 and minimally for COL1 (Figure 5b), which indicated repair by hyaline cartilage. Samples from transplantation group C stained weakly for COL2 and strongly for COL1 (Figure 5c), which indicated repair by both hyaline cartilage and fibrocartilage. Immunostaining with human‐specific vimentin antibody showed successful engraftment of human cells only in Group C (Figure 5c). No regenerated cartilage was detected in Groups D or E.

**Figure 6:**
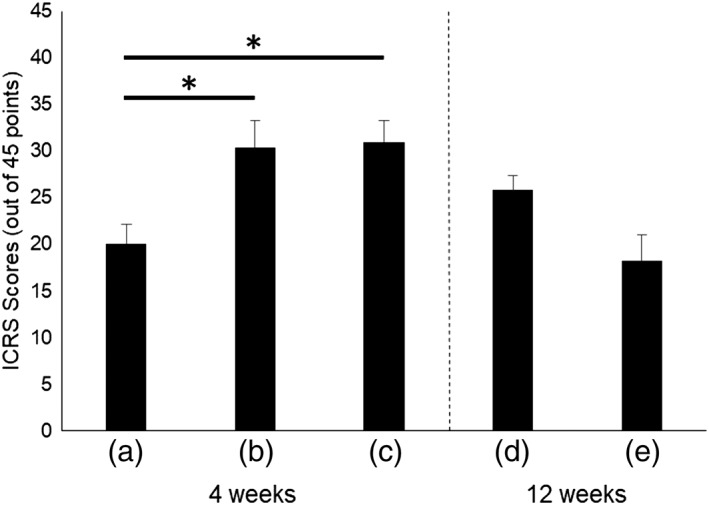
International Cartilage Repair Society (ICRS) scores in the treatment groups. (a) Group A: defect only, 4 weeks, 1.6 mg/kg/day of tacrolimus; (b) Group B: total knee arthroplasty (TKA) sheet, 4 weeks, 0.8 mg/kg/day; (c) Group C: TKA sheet, 4 weeks, 1.6 mg/kg/day; (d) Group D: defect only, 12 weeks, 1.6 mg/kg/day; and (e) Group E: TKA sheet, 12 weeks, 1.6 mg/kg/day. At 4 weeks, ICRS scores were significantly higher for groups B (^*^ *p* = 0.020) and C (^*^ *p* = 0.014) than for Group A. At 12 weeks, the scores did not differ significantly between Groups D and E (*p* = 0.07)

A modified version of the ICRS grading system was used to evaluate cartilage repair (Figure 6). At 4 weeks, the scores were significantly higher in transplantation groups B (30.4 ± 2.8, *p* = 0.020) and C (31.0 ± 2.2, *p* = 0.014) than in defect group A (20.1 ± 2.0). At 12 weeks, the scores did not differ significantly (*p* = 0.07) between transplantation group E (18.2 ± 2.8) and defect group D (25.8 ± 1.6).

## 4 DISCUSSION

Hyaline cartilage regeneration using chondrocyte sheets may provide an effective and long‐term treatment for OA. To ensure the safety and efficacy of this treatment using different cell sources, preclinical models are needed for evaluating human chondrocyte sheets directly. Such models will also be critical for evaluating differences associated with donor age, gender, health status, and other factors yet to be identified. In this study, we have shown the usefulness of a rabbit xenogeneic transplantation model for the direct evaluation of human chondrocyte sheets.

JW rabbits were immunosuppressed by tacrolimus at two different concentrations, 0.8 and 1.6 mg/kg/day, and human TKA sheets were transplanted into osteochondral defects. We verified that TKA sheets expressed fibronectin important to the adhesive properties of chondrocyte sheets and that they also produced TGFβ‐1 and MIA, which are known anabolic factors that may contribute to the regenerative effects. At 4 weeks and under immunosuppression of 1.6 mg/kg/day, successful engraftment of human chondrocytes, pain alleviation, and improvement in histological scores were observed. To our knowledge, this is the first study to provide clear evidence of the successful engraftment of human chondrocytes in the injured rabbit knee and to characterize the cartilage matrix produced by the transplanted chondrocytes. Immunostaining indicated repair by both hyaline cartilage and fibrocartilage, which may be indicative of the advanced age and OA nature of the adult chondrocytes used in this study. Muscle stiffness and muscle atrophy at the sites of tacrolimus injections were observed on both hind legs, but the results of the weight distribution ratios were comparable with those reported in our previous study (Ito et al., 2012).

At 4 weeks and under immunosuppression of 0.8 mg/kg/day, almost no engraftment of human chondrocytes was observed, but there was strong regeneration with hyaline cartilage as well as pain alleviation and improvement in histological scores. The observed regenerative effect may be attributed to the paracrine effect of humoral factors produced by chondrocyte sheets, as we reported previously (Hamahashi et al., 2015). The paracrine effect was also reported to be the major mode of action of cell sheet treatment of ischaemic cardiomyopathy in a porcine xenogeneic transplantation model (Kawamura et al., 2012; Kawamura et al., 2015). The rejection of transplanted cells may occur in parallel with the paracrine effect and may result in regeneration of hyaline cartilage by activated host cells even when no donor cells remain.

We also examined whether this model could be used to evaluate the remodelling of articular cartilage over the long term. We hypothesized that, after successful engraftment and matrix production, immunosuppression may be unnecessary. However, histological evaluation at 12 weeks after transplantation (i.e., 8 weeks after termination of immunosuppression) showed that immune rejection had occurred. The articular cartilage has long been considered a relatively immune‐privileged site, but recent findings have been inconsistent. For example, using porcine chondrocytes in a rabbit model, Ramallal et al. (2004) reported no immune rejection at 24 weeks in a xenogeneic transplantation study. However, delayed immune rejection was suggested in a similar study by another laboratory (Pei, Yan, Shoukry, & Boyce, 2010). Xenogeneic transplantation studies using human chondrocytes in minipigs (Niemietz et al., 2014) and human osteochondral biphasic composite constructs in rabbits (Jang, Lee, Park, Song, & Wang, 2013) have also reported immune rejection. Thus, most of the evidence suggests that immunosuppression is necessary for long‐term studies and that articular cartilage is not necessarily immune‐privileged in xenogeneic transplantation.

A key limitation of our study is that tacrolimus has been shown to reduce OA‐like responses and to protect cartilage matrix integrity in vitro and in vivo (Siebelt et al., 2014). These effects may complicate the interpretation of our results. Intramuscular administration of tacrolimus alone was insufficient for allowing the regeneration of articular cartilage in this rabbit model. However, tacrolimus may stimulate or modify the cartilage‐regenerating effect resulting from the transplantation of chondrocyte sheets. Further studies are needed to determine the extent to which transplanted cells may be affected.

Another limitation is that tacrolimus administration was accompanied by adverse events such as weight loss and self‐inflicted wounds. Self‐inflicted wounds and muscle loss increased the variability in the weight distribution ratio. Blood monitoring did not indicate kidney or liver failure, but these adverse events limited tacrolimus administration to 4 weeks in this study and would limit its use in longer studies. Differences in tacrolimus toxicity between rabbit species must also be considered in order to translate our results to other rabbit species. Severe tacrolimus toxicity was reported in the Dutch‐Belted rabbit, and a much lower dosage of 0.08 mg/kg/day has been suggested as feasible (Giessler, Gades, Friedrich, & Bishop, 2007). JW rabbits can tolerate 1.6 mg/kg/day, as first described by Ikebe et al. (1996) in bone xenogeneic transplantation, but the optimal concentrations need to be determined in further studies.

Chondrocyte sheets are unique in that the transplanted chondrocytes may survive over the long term in the recipient in addition to contributing to the regeneration of cartilage through a paracrine effect. Few clinical studies have tracked the fate of donor chondrocytes in humans. In several studies with fresh osteochondral allografts, donor chondrocytes were reported to be alive and active in the patients after 29 years (Jamali, Hatcher, & You, 2007). The same research group published another case report identifying, without exception, the engraftment of donor allograft cells in the location of the allografts after 3 years (Haudenschild, Hong, Hatcher, & Jamali, 2012). Although xenogeneic transplantation may not completely reproduce the results of allogeneic transplantation, a xenogeneic transplantation model that assesses both the paracrine effect and the characteristics of the engrafted chondrocytes is essential.

A rabbit xenogeneic transplantation model using JW rabbits with intramuscular administration of tacrolimus was feasible over a short span of 4 weeks. Ascertaining the efficacy of human chondrocyte sheets and other regenerative therapies for articular cartilage repair through xenogeneic transplantation of human cells is important for determining the in vivo characteristics of donor cells. We will use this preclinical model in the future to evaluate different cell sources and donor differences to ensure in vivo efficacy.

## CONFLICT OF INTEREST

M. S. is one of inventors on the patent (WO2006093151) submitted by the main applicant CellSeed Inc. for the manufacturing process of chondrocyte sheets. M. S. receives research funds from CellSeed Inc.
